# Tumor-associated NADH oxidase (tNOX)-NAD^+^-sirtuin 1 axis contributes to oxaliplatin-induced apoptosis of gastric cancer cells

**DOI:** 10.18632/oncotarget.14787

**Published:** 2017-01-21

**Authors:** Huei-Yu Chen, Hsiao-Ling Cheng, Yi-Hui Lee, Tien-Ming Yuan, Shi-Wen Chen, You-Yu Lin, Pin Ju Chueh

**Affiliations:** ^1^ Institute of Biomedical Sciences, National Chung Hsing University, Taichung, 40227, Taiwan; ^2^ Department of Surgery, Feng-Yuan Hospital, Ministry of Health and Welfare, Taichung 42055, Taiwan; ^3^ Graduate Institute of Basic Medicine, China Medical University, Taichung, 40402, Taiwan; ^4^ Department of Medical Research, China Medical University Hospital, Taichung, 40402, Taiwan; ^5^ Department of Biotechnology, Asia University, Taichung, 41354, Taiwan

**Keywords:** apoptosis, deacetylase, oxaliplatin, tumor-associated NADH oxidase (tNOX or ENOX2), sirtuin 1 (SIRT1)

## Abstract

Oxaliplatin belongs to the platinum-based drug family and has shown promise in cancer treatment. The major mechanism of action of platinum compounds is to form platinum–DNA adducts, leading to DNA damage and apoptosis. Accumulating evidence suggests that they might also target non-DNA molecules for their apoptotic activity. We explored the effects of oxaliplatin on a tumor-associated NADH oxidase (tNOX) in gastric cancer lines. In AGS cells, we found that the oxaliplatin-inhibited tNOX effectively attenuated the NAD^+^/NADH ratio and reduced the deacetylase activity of an NAD^+^-dependent sirtuin 1, thereby enhancing p53 acetylation and apoptosis. Similar results were also observed in tNOX-knockdown AGS cells. In the more aggressive MKN45 and TMK-1 lines, oxaliplatin did not inhibit tNOX, and induced only minimal apoptosis and cytotoxicity. However, the downregulation of either sirtuin 1 or tNOX sensitized TMK-1 cells to oxaliplatin-induced apoptosis. Moreover, tNOX-depletion in these resistant cells enhanced spontaneous apoptosis, reduced cyclin D expression and prolonged the cell cycle, resulting in diminished cancer cell growth. Together, our results demonstrate that oxaliplatin targets tNOX and SIRT1, and that the tNOX-NAD^+^-sirtuin 1 axis is essential for oxaliplatin-induced apoptosis.

## INTRODUCTION

Cisplatin belongs to a family of platinum-based compounds that has been used as a chemotherapeutic drug for many types of cancer [1]. However, because of unexpected side effects and drug resistance, other platinum-based compounds have been developed. One such drug, oxaliplatin, is a third-generation platinum compound that has shown promise in the treatment of colon and pancreatic cancers [1]. The major action mechanism of platinum compounds is considered to be their ability to bind DNA and form platinum–DNA adducts, leading to DNA damage and apoptosis [2]. In addition to their primary DNA targets, the platinum-based drug compounds are thought to target non-DNA molecules, thereby contributing to their pro-apoptotic effects and cytotoxicity [3, 4].

We previously identified a tumor-associated NADH oxidase (tNOX, ENOX2) that oxidizes NADH or hydroquinones to the oxidized NAD^+^ form, and demonstrated that it is involved in regulating cancer cell growth [5–8]. This activity was also detected in pooled sera from cancer patients but not healthy volunteers, making tNOX an attractive target for translational medicine research [9–11]. Interestingly, tNOX has shown potential as a target for various anti-cancer drugs, including capsaicin [12], EGCg [13], adriamycin (doxorubicin) [14, 15], phenoxodiol [16], and most recently the 4,11-diaminoanthra[2,3-b]furan-5,10-diones [17]. The therapeutic targeting of tNOX can inhibit cell growth and induce apoptosis in transformed cells but not in non-cancer cells [12, 13, 17], and forced depletion of tNOX has been shown to enhance the sensitivity of cells to various stresses [18–20]. The preference of tNOX for tumor cells over normal cells has been confirmed by both gain-of-function and loss-of-function approaches in cell culture systems [21, 22]. Intriguingly, anticancer drugs were found to transiently up-regulate tNOX expression, thereby enhancing migration and the epithelia-mesenchymal transition (EMT) [15, 23]; this may help explain the acquisition of drug resistance by cells subjected to these anticancer treatments. Together, these lines of evidence support the notion that tNOX is a potential therapeutic target, as it appears to govern cellular outcomes under a variety of stresses, from apoptosis to tumorgenesis.

To explore whether tNOX is targeted by oxaliplatin, we employed three lines of gastric cancer cells that differed in their levels of tNOX expression and aggressiveness. Our results suggest that oxaliplatin-induced apoptosis in AGS cells occurs through tNOX-induced alterations in the levels of NAD^+^/NADH and sirtuin 1 (SIRT1) deacetylase, and that these changes enhance p53 acetylation and apoptosis.

## RESULTS

### Oxaliplatin downregulates tNOX protein expression and induces apoptosis in AGS cells

**Figure 1:**
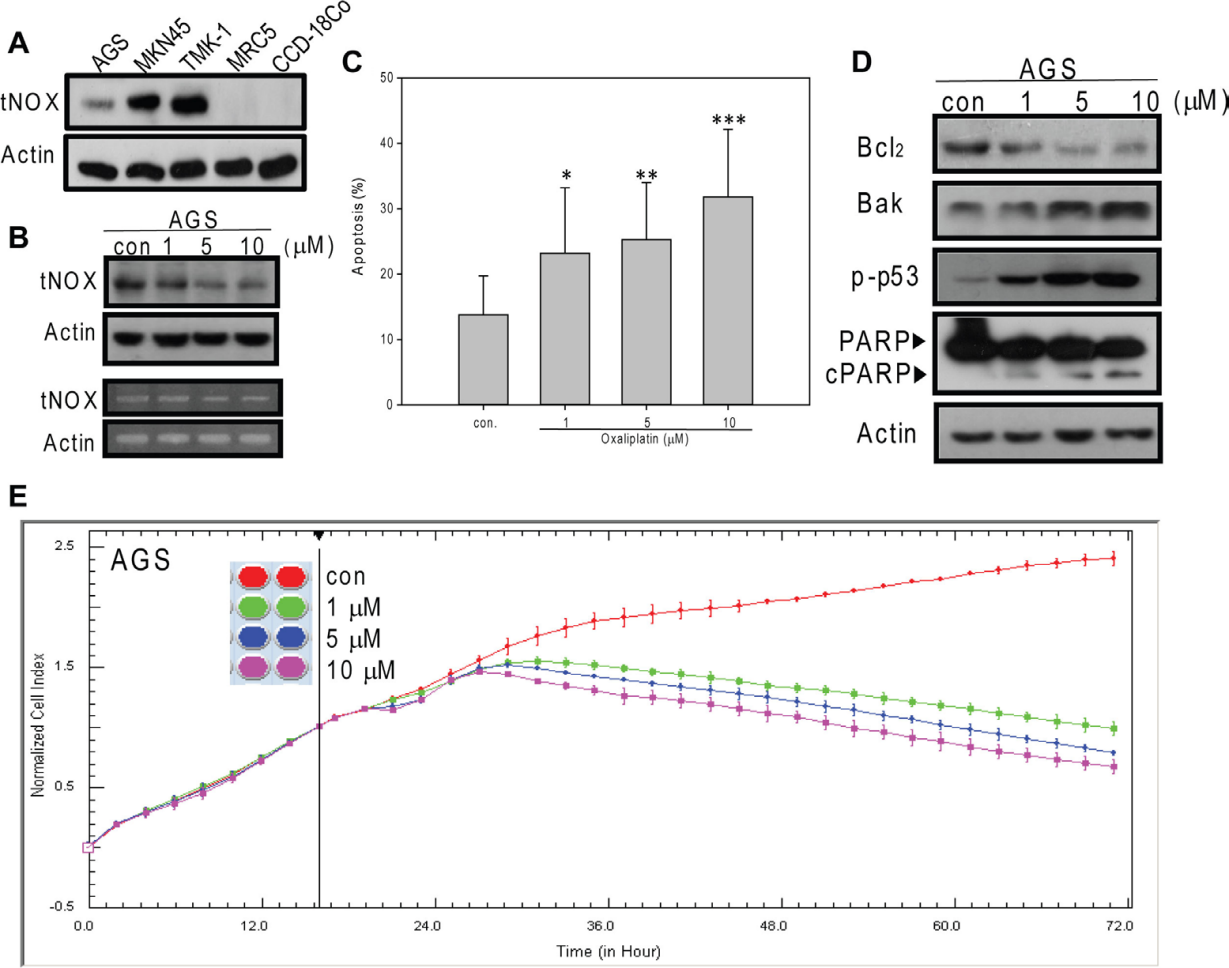
Oxaliplatin suppresses the growth of AGS cancer cells by inducing of apoptosis (**A**) Cell lysates were separated by SDS-PAGE and analyzed by Western blotting. (**B** and **D**) AGS cells were treated with oxaliplatin or ddH_2_O for 24 hours, and cell lysates were separated by SDS-PAGE and analyzed by Western blotting (B upper panel). tNOX mRNA levels were determined by RT-PCR (B lower panel). β-Actin was used as an internal control. Representative images are shown. (**C**) AGS cells were treated with oxaliplatin or ddH_2_O for 24 hours and the percentage of apoptotic cells was determined by flow cytometry. The results are presented as the percentage of apoptotic cells; the presented values (mean ± SEs) represent three independent experiments performed in at least triplicate (*p < 0.05, **p < 0.01, ***p < 0.001 for AGS cells treated with oxaliplatin vs. controls). (**E**) AGS cells were treated with or without oxaliplatin and cell growth was dynamically monitored using impedance technology. Normalized cell index values measured over 70 hours are shown.

We herein studied the effect of oxaliplatin on the tNOX protein and assessed the underlying mechanism in three cell lines derived from human stomach cancers: AGS (gastric adenocarcinoma), MKN45 (from the lymph node of a moderately differentiated stomach adenocarcinoma), and TMK-1 (from a poorly differentiated adenocarcinoma). We founded that the tNOX protein is expressed to varying degrees in all three gastric cancer lines, with AGS (wild type p53), which is the least aggressive line, showing a low level of tNOX expression (Figure 1A). Moreover, tNOX expression was not detected in non-cancer human lung tissue MRC-5 or colon CCD-18Co cells (Figure 1A). Oxaliplatin effectively reduced tNOX expression at both the transcriptional and translational levels (Figure 1B). Since stress-mediated tNOX downregulation is associated with apoptosis [18, 20], we analyzed apoptotic populations. Our results revealed that oxaliplatin induced significant apoptosis at 1 μM (Figure 1C), and that this was accompanied by downregulation of the anti-apoptotic protein, Bcl-2, and up-regulation of the pro-apoptotic responses of Bax expression, p53 activation, and caspase 3-directed PARP cleavage (Figure 1D). When dynamic cell growth was continuously monitored by cell impedance measurements, which are presented as cell index (CI) values, we found that oxaliplatin decreased the CI values in AGS cells, with the IC_50_ value calculated to be 0.6 μM (Figure 1E).

### The tNOX-mediated modulation of the NAD^+^ concentration and SIRT1 are involved in oxaliplatin-induced apoptosis

**Figure 2:**
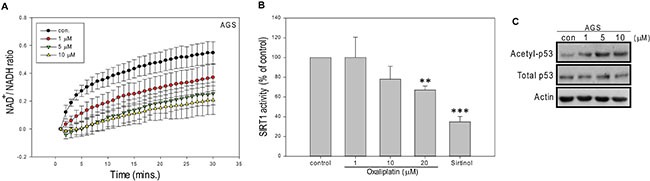
Oxaliplatin reduces NAD^+^/NADH ratio and SIRT1 activity in AGS cells (**A**) AGS cells were treated with ddH_2_O or oxaliplatin, and the NAD^+^ and NADH levels in cell extracts were quantified. The optical density at 450 nm was recorded and used to calculate the NAD^+^/NADH ratio. Values (mean ± SEs) were obtained from at least three independent experiments. (**B**) Samples were incubated with oxaliplatin or ddH_2_O, and the *in vitro* SIRT1 deacetylase activity was determined using a direct fluorescence assay kit. Sirtinol was used as a control. Values (means ± SEs) were obtained from three independent experiments performed in at least triplicate (**p < 0.01 and ***p < 0.001 for samples treated with oxaliplatin or sirtinol vs. controls). (**C**) AGS cells were treated with oxaliplatin or ddH_2_O for 24 hours, and cell lysates were separated by SDS-PAGE and analyzed by Western blotting. β-Actin was used as an internal control. Representative images are shown.

Next, we explored whether tNOX could be involved in the molecular mechanisms that govern oxaliplatin-mediated growth inhibition and apoptosis. As tNOX catalyzes the oxidation of reduced NADH to oxidized NAD^+^ [6, 7], we questioned whether oxaliplatin affects the intracellular levels of NAD^+^ and NADH. Indeed, we found that exposure of AGS cells to oxaliplatin at doses of 1 μM and above effectively decreased the intracellular NAD^+^/NADH ratio compared to that in control cells (Figure 2A), possibly due to the inhibition of tNOX activity. The NAD^+^/NADH ratio is an indicator of cellular redox balance, which affects many cellular pathways. The availability of NAD^+^ also modulates the activity of SIRT1, which acts as a deacetylase for both histone and non-histone proteins (including the tumor suppressor, p53) and affects many cellular responses [24]. We examined the effect of oxaliplatin on SIRT1 activity, and found that the *in vitro* activity of recombinant SIRT1 was significantly inhibited by 20 μM oxaliplatin, making its inhibitory power comparable to that of the well-defined SIRT1 inhibitor, sirtinol (25 μM) (Figure 2B). Interestingly, we observed an increase in p53 acetylation in cells treated with as little as 1 μM of oxaliplatin (Figure 2C). The SIRT1 activity requires NAD^+^ as a cofactor and we speculate that the tNOX-mediated decrease of NAD^+^ concentrations and oxaliplatin-induced down-regulation of tNOX together reduce SIRT1deacetylase activity *in vivo*, enhancing p53 acetylation and apoptosis. These results are consistent with a previous report demonstrating that SIRT1-mediated p53 deacetylation can negatively regulate stress-mediated apoptosis [25].

**Figure 3:**
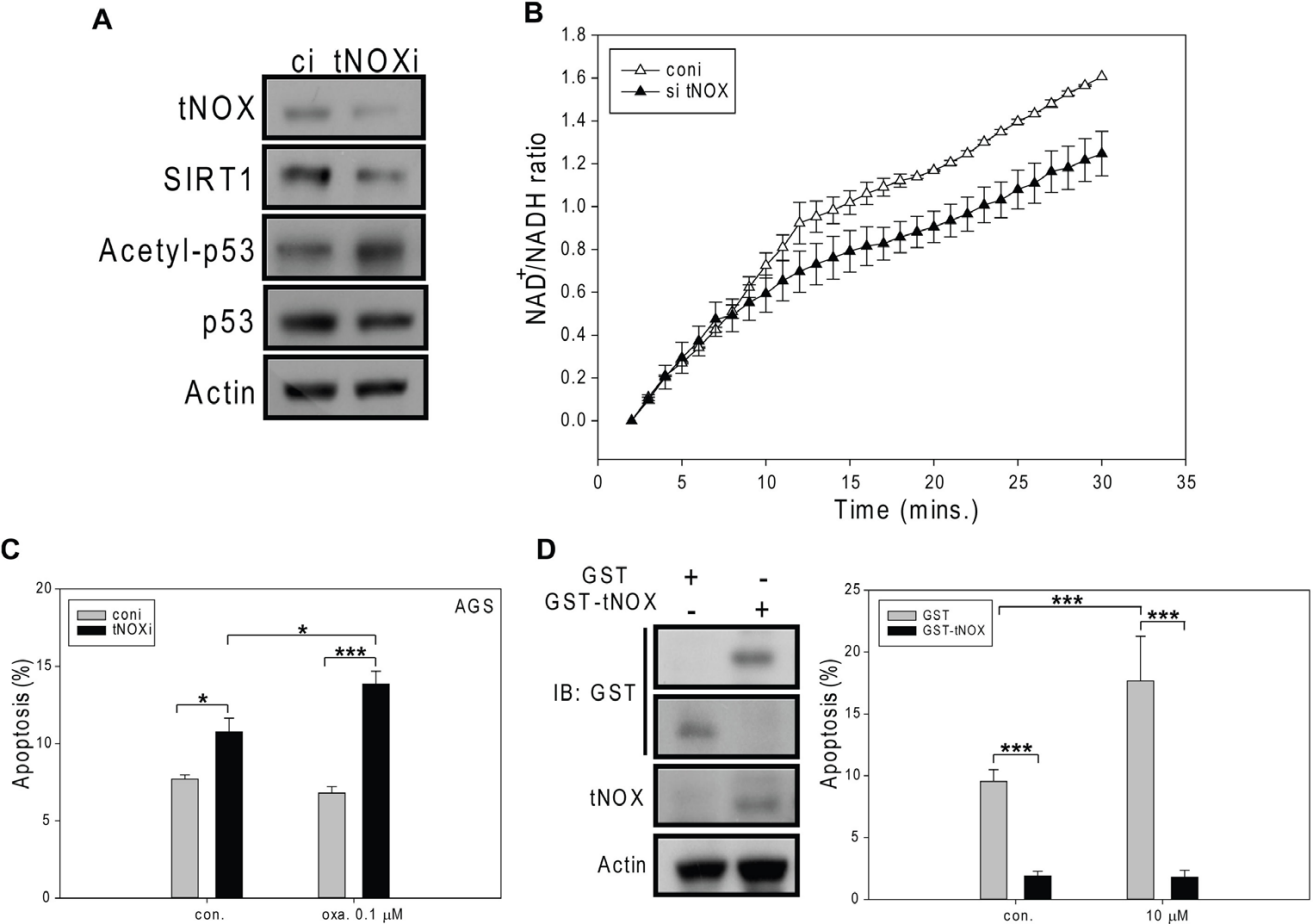
The effects of siRNA-mediated tNOX knockdown or tNOX overexpression on oxaliplatin-induced apoptosis in AGS cells tNOX was knockdown by RNA interference in AGS cells (**A**, **B**) and then these cells were treated with ddH_2_O or oxaliplatin for 24 hours, and the percentage of apoptotic cells was determined by flow cytometry (**C**). A, Cell lysates were separated by SDS-PAGE and analyzed by Western blotting. β-Actin was used as an internal control. Representative images are shown. B, NAD^+^ and NADH were quantified in extracts of control and tNOX-knockdown cells. The optical density at 450 nm was recorded and used to calculate the NAD^+^/NADH ratio. The presented values (mean ± SEs) represent at least three independent experiments. C, The percentage of apoptotic cells was determined by flow cytometry. The results are presented as the percentage of apoptotic cells; the presented values (mean ± SEs) represent at least three independent experiments (*p < 0.05 or ***p < 0.001 for tNOX-knockdown cells vs. controls). (**D**) AGS cells were transfected with GST or GST-tNOX wild type, and incubated for 48 hours. Overexpression of GST-tNOX was analyzed using GST or tNOX antibodies. β-actin was used as an internal control to monitor for equal loading. Representative images are shown. These cells were then treated with ddH_2_O or oxaliplatin for 24 hours, and the percentage of apoptotic cells was determined by flow cytometry. The results are presented as the percentage of apoptotic cells; the presented values (mean ± SEs) represent at least three independent experiments (***p < 0.001 for tNOX-overexpressing cells vs. controls).

We next used RNA interference (RNAi) to down-regulate tNOX expression in AGS cells. RNA interference targeting tNOX reduced expression of SIRT1 and this SIRT1 suppression was accompanied by enhanced p53 acetylation (Figure 3A). As expected, the intracellular NAD^+^/NADH ratio was decreased in these tNOX-knockdown AGS cells (Figure 3B). More importantly, apoptotic populations were significantly higher in tNOX-knockdown AGS control cells and tNOX-depletion also enhanced sensitivity of AGS cells to oxaliplatin-induced apoptosis at 0.1 μM compared to the controls (Figure 3C). On the other hand, overexpression of tNOX in AGS cells has reversed the oxaliplatin-mediated apoptosis, suggesting a pro-survival role of tNOX (Figure 3D).

### MKN45 and TMK-1 cells are less responsive to the apoptotic effect of oxaliplatin

**Figure 4:**
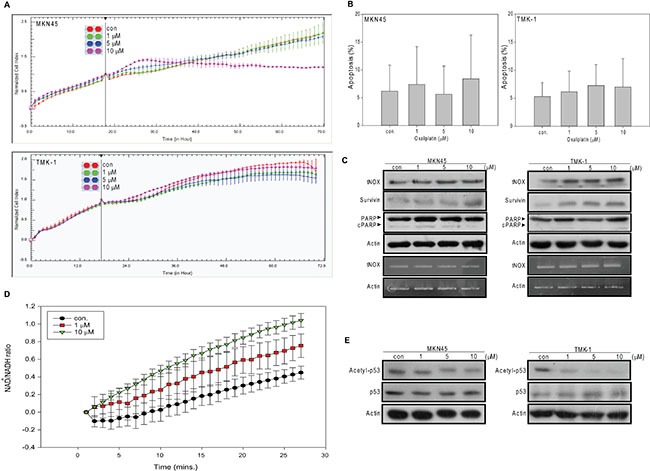
Oxaliplatin has less cytotoxic and apoptotic activity in MKN45 and TMK-1 cells than in AGS cells (**A**) Cell were treated with ddH_2_O or different concentrations of oxaliplatin and cell growth was dynamically monitored using impedance technology. Normalized cell index values measured over 70 hours are shown. (**B**) Cells were treated with oxaliplatin or ddH_2_O for 24 hours, and the percentage of apoptotic cells was determined by flow cytometry. The results are presented as the percentage of apoptotic cells; the presented values (mean ± SEs) represent at least three independent experiments performed in at least triplicate (no significant between-group difference was observed). (**C** and **E**) Cells were treated with oxaliplatin or ddH_2_O for 24 hours and cell lysates were separated by SDS-PAGE and analyzed by Western blotting (C upper panel). tNOX mRNA levels were determined by RT-PCR (C lower panel). β-Actin was used as an internal control. Representative images are shown. (**D**) TMK-1 cells were treated with ddH_2_O or oxaliplatin, cell extracts were prepared, and the levels of NAD^+^ and NADH were quantified. The optical density at 450 nm was recorded and used to calculate the NAD^+^/NADH ratio. The presented values (mean ± SEs) represent at least three independent experiments.

We next examined the effect of oxaliplatin on the more aggressive gastric cancer lines, MKN45 (wild type p53) and TMK-1 (mutant p53). Interestingly, oxaliplatin exhibited much less cytotoxicity toward these two lines, with our cell impedance measurements yield IC_50_ values of 18.2 and 97.8 μM, respectively (Figure 4A). Consistent with this, minimal induction of apoptosis was observed in treated cells, even among those exposed to 10 μM of oxaliplatin regardless of their p53 status (Figure 4B). Consistent with the tumor-promoting role of tNOX suggested by many previous studies, oxaliplatin-treated MKN45 and TMK-1 cells showed no down-regulation of tNOX at both translational and transcriptional levels (in fact, some up-regulation of tNOX was observed in TMK-1 cells), increased expression of survivin (an anti-apoptotic factor), and no evidence of caspase 3-directed PARP cleavage (Figure 4C). Unlike our finding in AGS cells, the intracellular NAD^+^/NADH ratio in TMK-1 cells was increased by oxaliplatin (Figure 4D), possibly due to the enhancement of tNOX. Also in contrast to our findings in AGS cells, oxaliplatin caused a decrease in p53 acetylation in both cell lines (Figure 4E). This suggests that, in these aggressive cell lines, oxaliplatin increased the intracellular NAD^+^/NADH ratio to enhance SIRT1 activity and p53 deacetylation, and thus, did not trigger apoptosis, at least in functional p53-expressing MKN45 cells.

**Figure 5:**
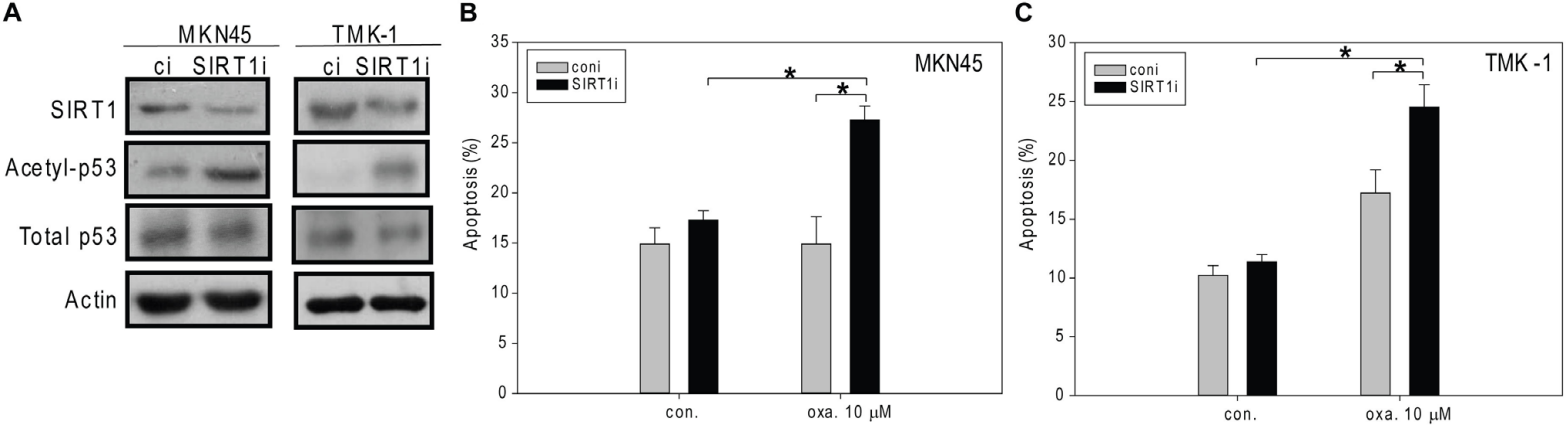
The siRNA-mediated knockdown of SIRT1 enhances oxaliplatin-induced apoptosis in MKN45 and TMK-1 cells (**A**, **B**) SIRT1 was knockdown by RNA interference (A) and then these cells were treated with ddH_2_O or oxaliplatin for 24 hours, and the percentage of apoptotic cells was determined by flow cytometry (B, **C**). A, Cell lysates were separated by SDS-PAGE and analyzed by Western blotting. β-Actin was used as an internal control. Representative images are shown. B, The results are presented as the percentage of apoptotic cells; the presented values (mean ± SEs) represent at least three independent experiments (*p < 0.05 for SIRT1-knockdown cells treated with 10 mM oxaliplatin vs. controls).

To further examine the role of SIRT1 in oxaliplatin-induced apoptosis, we used RNAi to down-regulate SIRT1 expression in MKN45 and TMK-1 cells (Figure 5A). In both cell lines, transfection of SIRT1-targeting small-interfering RNA (siRNA) significantly enhanced the levels of p53 acetylation (Figure 5A). Significantly enhanced apoptosis was also observed in SIRT1-knockdown MKN45 cells exposed to 10 μM oxaliplatin (Figure 5B). Interestingly, oxaliplatin induced marked apoptosis in SIRT1-depleted TMK-1 cells compared to the controls regardless of the presence of mutant p53 (Figure 5C). Consistent with the findings by Kim et al. [26], our results support the idea that suppression of SIRT1 enhanced the sensitivity of cells to stress-induced apoptosis.

### RNAi-mediated tNOX downregulation enhances spontaneous apoptosis and prolongs cell cycle

**Figure 6:**
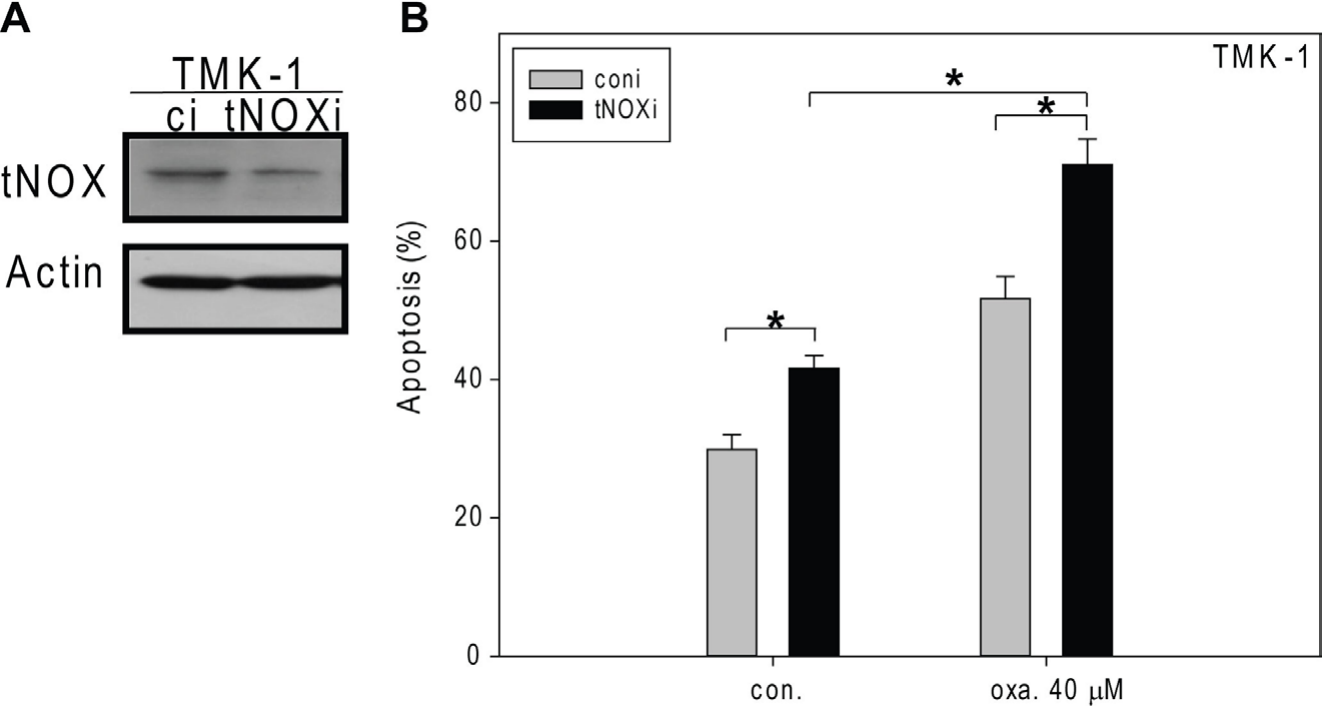
The effects of siRNA-mediated tNOX knockdown on oxaliplatin-induced apoptosis in TMK-1 cells (**A**, **B**) tNOX was knockdown by RNA interference (A) and then these cells were treated with ddH_2_O or oxaliplatin for 24 hours, and the percentage of apoptotic cells was determined by flow cytometry (B). A, Cell lysates were separated by SDS-PAGE and analyzed by Western blotting. β-Actin was used as an internal control. Representative images are shown. B, The results are presented as the percentage of apoptotic cells; the presented values (mean ± SEs) represent at least three independent experiments (*p < 0.05 for tNOX-knockdown cells vs. controls).

We further utilized RNAi to knock down tNOX in TMK-1 cells, which were the most aggressive cells tested herein (Figure 6A). Interestingly, tNOX-depleted cells exhibited a higher level of spontaneous apoptosis than control cells but not to the significant level (Figure 6B). tNOX-knockdown cells exposed to 40 μM oxaliplatin indeed showed considerably enhanced apoptosis compared to scrambled siRNA-transfected cells (Figure 6B). These results indicate that tNOX is important for cancer cell survival, and downregulation of tNOX enhances the sensitivity of resistant TMK-1 cells to oxaliplatin-induced apoptosis.

**Figure 7:**
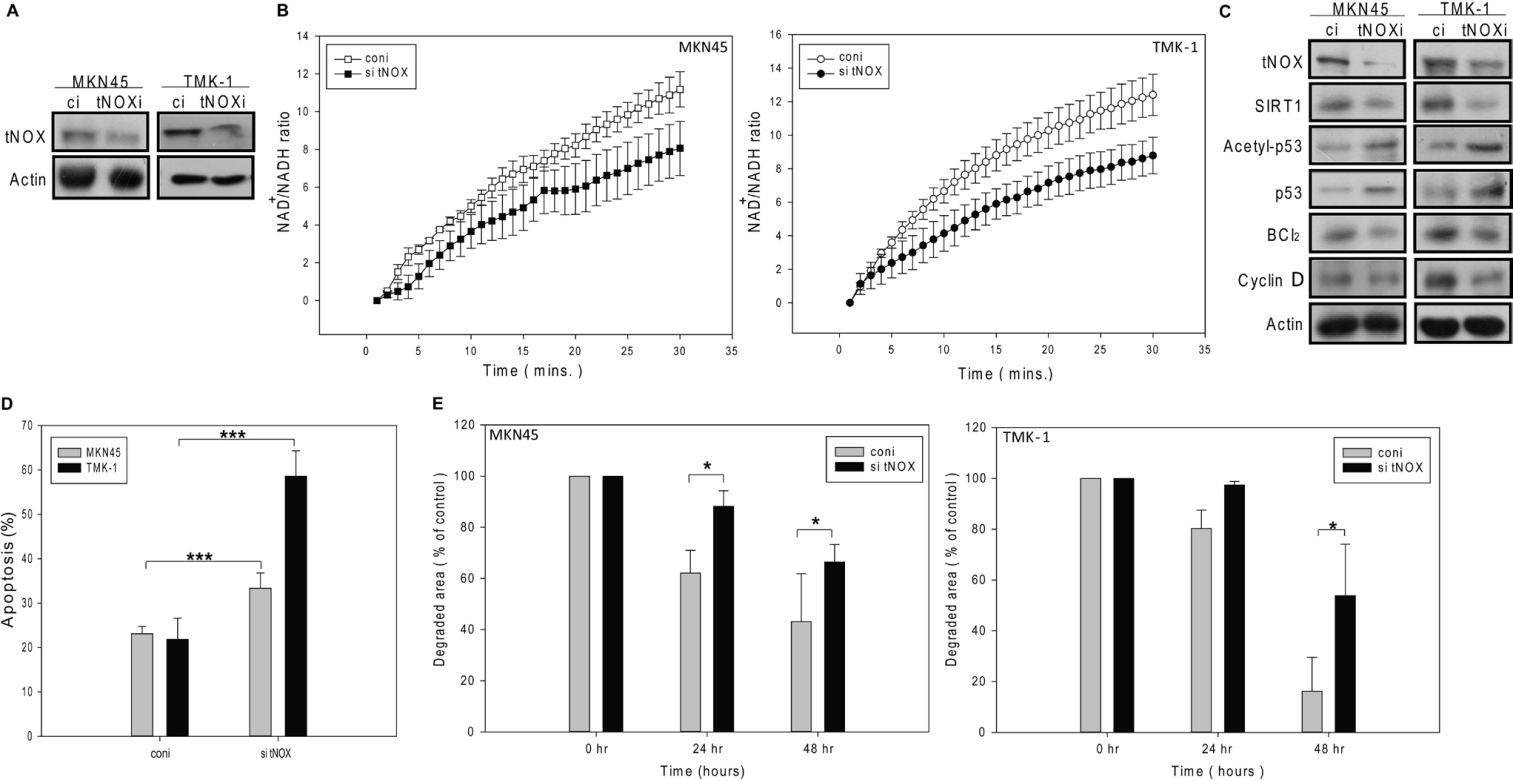
The siRNA-mediated knockdown of tNOX reduces the cancer phenotypes in MKN45 and TMK-1 cancer cells (**A** and **C**) Cell lysates were separated by SDS-PAGE and analyzed by Western blotting. β-Actin was used as an internal control. Representative images are shown. (**B**) NAD^+^ and NADH were quantified in extracts of control and tNOX-knockdown cells. The optical density at 450 nm was recorded and used to calculate the NAD^+^/NADH ratio. The presented values (mean ± SEs) represent at least three independent experiments. (**D**) The percentage of apoptotic cells was determined by flow cytometry. The results are presented as the percentage of apoptotic cells; the presented values (mean ± SEs) represent at least three independent experiments (***p < 0.001 for tNOX-knockdown cells vs. controls). (**E**) Cell division was analyzed by CMFDA staining of tNOX-knockdown and control cells. The presented values (mean ± SEs) represent at least three independent experiments (*p < 0.05 for tNOX-knockdown cells vs. controls).

Next, we knocked down tNOX expression in both MKN45 and TMK-1 cells (Figure 7A), and examined various phenotypes. In these tNOX-depleted cells, the intracellular NAD^+^/NADH ratio was significantly attenuated compared to the scrambled siRNA-transfected controls (Figure 7B). Moreover, SIRT1 expression was attenuated, p53 acetylation was enhanced, and the pro-survival factor, Bcl-2, was down-regulated, in the knockdown cells (Figure 7C). These findings are all consistent with the notion that SIRT1 and tNOX possess tumor-promoting properties. Ultimately, tNOX-knockdown also increased spontaneous apoptosis (Figure 7D). Cell cycle analysis revealed a lack of cell cycle arrest in knockdown cells (data not shown); instead, staining with CMFDA (a tracking dye for cell division) showed that tNOX knockdown increased the doubling time and prolonged cell cycle progression (Figure 7E). We also observed a decrease in the protein expression of cyclin D, an essential cell cycle regulator (Figure 7C). These data suggest that the tNOX protein acts as a pro-tumor factor by controlling the intracellular redox environment, which is important to the regulation of cell apoptosis and proliferation.

## DISCUSSION

In this study, we investigated the apoptotic effect of oxaliplatin on three different lines of gastric cancer cell lines (AGS, MKN45, and TMK-1 cells), focusing on potential non-DNA molecular targets. We found that the responsiveness of these cell lines toward oxaliplatin was reflected in the ability of tNOX to induce changes in the levels of NAD^+^ and SIRT1 deacetylase activity, which appeared to play influential role in oxaliplatin-induced apoptosis. In the oxaliplatin-sensitive AGS cell line, oxaliplatin repressed the tNOX-induced NAD^+^ level and SIRT1 deacetylase activity, thereby enhancing p53 acetylation and apoptosis. In contrast, oxaliplatin had much smaller inhibitory effects and triggered little apoptosis in MKN45 and TMK-1 cells, which are known to be relatively resistant to this agent. However, the depletion of either tNOX or SIRT1 sensitized TMK-1 cells to oxaliplatin-induced apoptosis, demonstrating their importance in this pathway.

One significant finding of this study is that SIRT1 plays a regulatory role in oxaliplatin-induced apoptosis. SIRT1, which is a well-studied member of the sirtuin protein family, requires NAD^+^ as cofactor to catalyze the deacetylation of histone and nonhistone proteins [27, 28]. SIRT1 is known to regulate a host of cellular events (e.g., lifespan and metabolism) through its deacetylase activity. However, its role in tumorgenesis is somewhat controversial, as some reports have indicated that it acts as a tumor promoter, while others have found it has tumor suppressor function [29–31]. In the present study, we provide additional evidence for the tumor-promoting function of SIRT1 by showing that oxaliplatin inhibits the growth of AGS cells via decreased SIRT1 activity, which enhances p53 acetylation and apoptosis.

In its role as a regulator of many metabolic reactions, NAD^+^ has been proposed to act through members of the SIRT family [32]. In this regard, the intracellular NAD^+^/NADH level and SIRT1 activity must be well maintained. Here, we suggest that tNOX can contribute to regulating the intracellular NAD^+^/NADH ratio and SIRT1 activity. We further suggest that the ability of oxaliplatin to induce apoptosis relates to its ability to inhibit tNOX and decrease the intracellular NAD^+^/NADH ratio. Thus, apoptosis in association with downregulation of tNOX and reduced NAD^+^ level were observed in AGS gastric cancer cells, whereas minimum apoptosis concurrent with a lack of tNOX downregulation and increased in NAD^+^/NADH ratio were noticed in MKN45 and TMK-1 metastatic gastric carcinoma lines. The results of our RNAi experiments in all three cell lines demonstrate that tNOX knockdown attenuates the intracellular NAD^+^/NADH ratio (Figure 3B and 7B), supporting the notion that tNOX contributes to maintaining the cellular NAD^+^/NADH redox status. These findings are consistent with those of Liang et al., who demonstrated that inhibition of SIRT1-mediated p53 deacetylation can suppress lung adenocarcinoma cells *in vitro* and *in vivo* [33]. Here, we explored further the mechanism underlying this regulation and show for the first time that the tNOX-modulated NAD^+^/NADH ratio-SIRT1-p53 axis contributes to oxaliplatin-induced apoptosis and cell growth suppression in AGS cells (Figures 1 and 2). However, our data from TMK-1 (p53 mutant) cells suggested that other downstream targets of SIRT1 might be involved in oxaliplatin-induced apoptosis, such as FOXO3 [34].

Oxaliplatin treatment is frequently associated with drug resistance, but numerous studies have demonstrated that the sensitivity of cells to oxaliplatin-induced cytotoxicity (and thus the therapeutic performance of this agent) can be improved by the knockdown of specific proteins, including Nrf2 [35], FOXM1/Mcl-1 [36], and Oc4, which acts through STAT3/survivin pathway [37]. There appears to be a positive relationship between tNOX expression and cancer phenotypes, including increased cell proliferation, migration and EMT [7, 15, 18, 22, 23, 38]. In this study, we show that oxaliplatin triggers apoptosis by down-regulating tNOX at both the translational and transcriptional levels in AGS cells (Figure 1). Oxaliplatin exhibits much less cytotoxicity against the more aggressive and oxaliplatin-resistant gastric cancer lines, MKN45 and TMK-1, but depletion of tNOX in these cells increased their susceptibility to the drug (Figures 4 and 6). The different effects of oxaliplatin appear to reflect its ability to inhibit tNOX expression in the three tested cell lines. Interestingly, tNOX depletion attenuated SIRT1 expression in all three cell lines (Figure 3A and 7C). As the knockdown of tNOX activates p53 (Figure 7C), we speculate that this effect on SIRT1 might reflect the ability of p53 to act as a transcription factor for microRNA-34a, which negatively regulates the SIRT1 protein [39–41].

Taken together, our results show that oxaliplatin mediates diverse inhibitory effects on the growth of gastric cancer cells, and demonstrate that tNOX knockdown sensitizes resistant gastric cancer cells to oxaliplatin-induced apoptosis. Furthermore, we provide evidence suggesting that the tNOX-induced modulation of the NAD^+^-SIRT1 axis is important to oxaliplatin-induced apoptosis. These findings show that tNOX plays an essential function in growth regulation and survival of gastric cancer cells, and may provide a rational framework for the further development of tNOX inhibitors as a novel class of antitumor agents.

## MATERIALS AND METHODS

### Materials

Fetal bovine serum (FBS) and penicillin/streptomycin were obtained from Gibco/BRL Life Technologies (Grand Island, NY, USA). The anti-Bak, anti-PARP, anti-Bcl-2, anti-p53, anti-phospho-p53, anti-acetyl-p53, anti-SIRT1, and anti-cyclin D1 antibodies were purchased from Cell Signaling Technology, Inc. (Beverly, MA, USA). The anti-b-actin antibody was from Millipore Corp. (Temecula, CA, USA). The antisera to tNOX used in Western blot analyses were generated as described previously [22]. The 3,3′-Dihexyloxacarbocyanine iodide [DiOC6(3)] was purchased from Calbiochem (San Diego, CA, USA). The anti-mouse and anti-rabbit IgG antibodies and other chemicals were purchased from the Sigma Chemical Company (St. Louis, MO, USA), unless specified otherwise.

### Cell culture and transfection

The AGS (gastric adenocarcinoma, p53 wild-type), MKN45 (from the lymph node of a moderately differentiated stomach adenocarcinoma, p53 wild-type), and TMK-1 (from a poorly differentiated adenocarcinoma, mutant p53) cell lines, which were all derived from human stomach cancers, were kindly provided by Dr. Chun-Ying Wu (Department of Gastroenterology, Taichung Veterans General Hospital, Taiwan). The cells were grown in RPMI-1640 medium supplemented with 10% FBS, 100 units/mL penicillin and 50 μg/mL streptomycin at 37°C in a humidified atmosphere of 5% CO_2_ in air, with replacement of the medium every 2–3 days. The experimental groups were treated with different concentrations of oxaliplatin dissolved in ddH_2_O, and the controls were treated with the same volume of ddH_2_O.

ON-TARGETplus tNOX (ENOX2) siRNA and negative control siRNA were purchased from Thermo Scientific, Inc. (Grand Island, NY) and SignalSilence Sirt1 siRNA I and control siRNA were from Cell Signaling Technology, Inc. (Beverly, MA, USA). Briefly, cells were seeded in 10-cm dishes, allowed to attach overnight, and then transfected with tNOX/Sirt1 siRNA and control siRNA using Lipofectamin RNAiMAX Reagent (Life Technologies, Grand Island, NY, USA) according to the manufacturer's instructions. Cells were also transiently transfected with tNOX-GST or GST (control) using the jetPEI transfection reagent according to the manufacturer's protocol (Polyplus-transfection SA, Illkirch Cedex, France).

### Continuous monitoring of cell impedance

For continuous monitoring of changes in cell growth, cells (7.5 × 10^3^ cells/well) were seeded onto E-plates and incubated for 30 min at room temperature. The E-plates were placed onto a Real-Time Cell Analysis (RTCA) station (Roche, Germany) and the cells were grown overnight before being exposed to oxaliplatin or ddH_2_O. Cell impedance was measured every hour for a total of 70 hours, as previously described [42], and defined by the cell index (CI) = (Z_i_ − Z_0_) [Ohm]/15[Ohm], where Z_0_ is background resistance and Z_i_ is the resistance at an individual time point. A normalized cell index was determined as the cell index at a certain time point (CI_ti_) divided by the cell index at the normalization time point (CI_nml_time_).

### Apoptosis determination

Apoptosis was measured using an Annexin V-FITC apoptosis detection kit (BD Pharmingen, San Jose, CA, USA). Cells cultured in 6-cm dishes were trypsinized, collected by centrifugation, washed, resuspended in 1× binding buffer, and stained with annexin V-FITC, as recommended by the manufacturer. Cells were also stained with propidium iodide (PI) to detect necrosis or late apoptosis. The distributions of viable (FITC/PI double-negative), early apoptotic (FITC-positive), late apoptotic (FITC/PI double-positive), and necrotic (PI-positive/FITC-negative) cells were analyzed using a Beckman Coulter FC500 flow cytometer. The results are expressed as a percentage of total cells.

### Measurement of intracellular NAD*^+^*/NADH ratio

The oxidized and reduced forms of intracellular nicotinamide adenine dinucleotide were determined using an NADH/NAD Quantification Kit (BioVision Inc. Milpitas, CA) as described by the manufacturer. Briefly, 2 × 10^5^ cells were washed with cold PBS, pelleted, and extracted by two freeze/thaw cycles with 400 μl of NADH/NAD^+^ extraction buffer. Samples were vortexed and centrifuged at 14,000 rpm for 5 min. The extracted NADH/NAD^+^ supernatant (200 μl) was transferred to a microcentrifuge tube, heated to 60°C for 30 min (to decompose NAD^+^ but not NADH), and then placed on ice. The samples were then centrifuged and transferred to a multiwall-plate. Standards and a NAD^+^ cycling mix were prepared according to the manufacturer's protocol. The reaction mix (100 μl) was distributed to each well containing NADH standards and samples, and the plates were incubated at room temperature for 5 minutes to convert NAD^+^ to NADH. The provided NADH developer solution was added to each well, and plates were incubated at room temperature for 15 or 30 minutes. The reaction was stopped with 10 μl of stop solution per well, and absorbance was measured at 450 nm.

### Measurement of SIRT1 deacetylase activity *in vitro*

SIRT1 deacetylase activity was determined using a SIRT1 direct Fluorescent Screening Assay Kit according to the manufacturer's protocol (Cayman Chemical Company, Ann Arbor, MI). Briefly, the substrate solution was prepared by adding 240 μl of NAD^+^ solution and 850 μl of diluted assay buffer to 15 μl of the p53 peptide Arg-His-Lys-Lys(εacetyl)-AMC, to yield a final concentration of 125 mM peptide (substrate) and 3 mM of NAD^+^. The background was determined in wells containing 30 μl of assay buffer and 5 μl of solvent. The maximal initial activity (defined as 100%) was determined in wells containing 5 μl of diluted human recombinant SIRT1, 25 μl of assay buffer, and 5 μl of solvent. To measure oxaliplatin-induced changes in SIRT1 activity, 5 μl of oxaliplatin was added to wells containing 25 μl of assay buffer and 5 μl of diluted human recombinant SIRT1. Reactions were initiated by adding 15 μl of substrate solution to each well. The plate was then covered and incubated on a shaker for 45 min at room temperature. The reactions were stopped by adding 50 μl of stop/developing solution to each well and incubating the plate for 30 minutes at room temperature. Plates were read in a fluorimeter using excitation wavelengths of 350–360 nm and emission wavelengths of 450–465 nm.

### Cell division assay

RNA interference-targeted tNOX knockdown cells and negative control siRNA-treated cells were labeled by incubating with 5 μM CellTracker Green CMFDA (5-chloromethylfluorescein diacetate; Molecular Probes, Eugene, OR, USA) in fresh medium for 45 minutes. After treatment, the cells were washed with PBS and trypsinized, and cell division was assessed by flow cytometry as previously described [43].

### Western blot analysis

Cell extracts were prepared in lysis buffer containing 20 mM Tris-HCl pH 7.4, 100 mM NaCl, 5 mM EDTA, 2 mM phenylmethylsulfonyl fluoride (PMSF), 10 ng/ml leupeptin, and 10 μg/ml aprotinin). Equal amounts of extracted proteins (40 μg) were resolved SDS-PAGE and transferred to nitrocellulose membranes (Schleicher & Schuell, Keene, NH, USA). The membranes were blocked with nonfat milk solution for an hour, and then washed and probed with the appropriate primary antibody. The membranes were rinsed with Tris-buffered saline containing 0.1% Tween 20, incubated with horseradish peroxidase-conjugated secondary antibody for 1 hour, rinsed again, and developed using enhanced chemiluminescence (ECL) reagents (Amersham Biosciences, Piscataway, NJ, USA).

### Reverse transcriptase-polymerase chain reaction (RT-PCR)

Total RNA from gastric cancer cells was isolated using the TRIzol reagent (GIBCO, Carlsbad, CA, USA). First strand cDNA was synthesized from 1 μg of total RNA using Superscript II (Life Technologies, Rockville, MD, USA). The following primers sets were used for PCR amplifications: tNOX, 5′-GAAGTGTGATGCCGATAACAG -3′′ (sense) and 5′-AGTACTAGAGCCCAGGCGAA-3′ (antisense); and b-actin, 5′-ACTCACCTTGGTGGTGCATA-3′ (sense) and 5′-ACACCTTGATGGGAAAGGTGG-3′ (antisense). The reaction conditions consisted of 30 cycles of 95°C for 30 seconds, 55°C for 30 seconds, and 72°C for 1 minute, followed by a final extension of 5 minutes at 72°C. The obtained PCR produces were resolved by 1.4% agarose gels electrophoresis and visualized by ethidium bromide staining.

### Statistics

All data are expressed as the means ± SEs of three or more independent experiments. Between-group comparisons were performed using one-way analysis of variance (ANOVA) followed by an appropriate post-hoc test. A value of *p* < 0.05 was considered to be statistically significant.
